# Interleukin-1 receptor antagonist inhibits angiogenesis in gastric cancer

**DOI:** 10.1007/s10147-018-1242-2

**Published:** 2018-01-17

**Authors:** Zhenqiang Gong, Jiachi Ma, He Su, Tiankang Guo, Hui Cai, Quan Chen, Xiaodan Zhao, Jianbo Qi, Jianbo Du

**Affiliations:** 1 Ningxia Medical University, Yinchuan, 750000 China; 2 Department of General Surgery, Gansu Provincial People’s Hospital, 204 Dong Gang West Road, Lanzhou, 730000 Gansu China; 3 Gansu University of Traditional Chinese Medicine, Lanzhou, 730000 China

**Keywords:** Interleukin-1 receptor antagonist, Angiogenesis, Gastric cancer

## Abstract

**Background:**

Interleukin-1 alpha (IL-1α) plays an important role in tumorigenesis and angiogenesis of gastric cancer. The interleukin-1 receptor antagonist (IL-1RA) inhibits IL-1 selectively and specifically through IL-1R type I (IL-1RI). However, the underlying mechanism by which IL-1RA modulates the interactions of tumor cells and their micro-environment is poorly understood. We have evaluated the role of IL-1RA in the metastatic process as well as the mutual or reciprocal actions between gastric cancer cells and stromal cells.

**Materials and methods:**

The expressions of IL-1α, vascular endothelial growth factor (VEGF), and IL-1RI mRNA were determined by reverse transcriptase-PCR. The regulatory effect of IL-1RA on the secretion of VEGF in human gastric cancer cells and human umbilical vein endothelial cells (HUVECs) was detected by enzyme-linked immunosorbent assay. The effect of IL-1RA on metastatic potential was evaluated using proliferation, invasion, and angiogenesis assays, respectively, including in vitro co-culture system models consisting of tumor cells and stromal cells that were used to detect invasion and angiogenesis.

**Results:**

Interleukin-1α mRNA was detected in the higher liver metastatic gastric cell line MKN45. IL-1α protein was expressed in MKN45 cells and in HUVECs. VEGF mRNA and protein were detected in the three gastric cancer cell lines (MKN4, NUGC-4, and AGS). Levels of VEGF secreted by gastric cancer cells and HUVECs appeared to be reduced through the action of IL-1RA via IL-1RI in a dose-dependent manner (*P* < 0.01). IL-1RA significantly inhibited the proliferation and migration of HUVECs (*P* < 0.01) and tube formation by HUVECs (*P* < 0.01), both in a dose-dependent manner. Compared with HUVECs grown without cancer cells (control) or with NUGC-4 cells, tube formation by HUVECs was significantly enhanced by co-culture with MKN45 cells (*P* < 0.01). The enhanced tube formation in the presence of MKN45 cells was inhibited by the addition of IL-1RA (*P* < 0.01).

**Conclusions:**

The IL-1RA downregulated the metastatic potential of gastric cancer through blockage of the IL-1α/VEGF signaling pathways. IL-1RA has the potential to play a role in the treatment of gastric cancer.

## Introduction

Interleukin-1 (IL-1) is a pro-inflammatory chemokine that interacts with specific membrane receptors on tumor cell surfaces and affects the proliferation and differentiation of tumor cell survival [1, 2]. The IL-1 family includes IL-1 alpha (IL-1α), IL-1 beta (IL-1β), and the IL-1 receptor antagonist (IL-1RA). Although IL-1α and IL-1β are derived from different genes, they are functionally similar, and both bind to the same receptor and exhibit similar biological activities [3]. IL-1α is localized in the cytosol or cell membrane that regulates the intracellular environment [4]. It regulates the expression of pro-metastatic genes, such as those of the matrix metalloproteinase family [5], CD44 [6], a multifunctional cell adhesion molecule, and c-MET, a tyrosine-protein kinase [7], and of activators of transcription factors, such as nuclear factor kappa B and activation protein-1 [8]. It also promotes tumor growth and metastasis via enhanced process of angiogenesis through its regulation of the expression of angiogenic factors, such as vascular endothelial growth factor (VEGF) [9–11].

IL-1RA is a naturally occurring anti-inflammatory molecule that shares homology with IL-1α and IL-1β. It binds to IL-1 receptor type I (IL-1RI) without delivering an activation signal; rather, it competitively antagonizes the binding of IL-1α and IL-1β to IL-1RI through its own binding to the receptor, possibly by changing the molecular conformation of the receptor. As such, IL-1RA is a physiologically active inhibitor of IL-1 [12]. IL-1RA also inhibits the expression levels of IL-6 and IL-8 in pancreatic cancer [13, 14] and of VEGF in gastric cancer cell [15–18]. In a murine model, administered exogenous IL-1RA inhibits hepatic metastases and its block accelerates tumor cell growth. The inhibition of IL-1 using micro-encapsulated genetically engineered cells that constitutively produce IL-1RA and which have been implanted in mice inhibits tumor angiogenesis and growth [19]. IL-1RA inhibits xenograft growth in IL-1-producing tumors but without direct anti-proliferative effects in vitro [20]. IL-1RA is safe when injected into the human body [21] and has been approved as a clinical medication that is effective in treating the signs and symptoms of rheumatoid arthritis [22].

VEGF is a key regulator of physiological angiogenesis in embryonic development, skeletal growth, and reproductive function, of which vascular permeability is one of its most important functions. VEGF is the prototypical angiogenic stimulated molecule that has been implicated in several steps throughout the angiogenic process [23]. Elevated levels of this cytokine are correlated with worse clinical outcomes in patients with a solid malignant tumor, including those of the breast [24], lung [25], colorectum [26], liver [27], and bladder [28]. VEGF is unique among angiogenic growth factors because it disrupts the endothelial barrier function, possibly contributing to the ability of tumor cells to break through the basement membrane and continue the infiltration of interstitial tissue [29]. It has been shown that increases in VEGF expression is directly proportional to increases in tumor growth and metastasis, as promoted by angiogenesis and increasing vascular permeability. The role of VEGF in angiogenesis is further confirmed by the effects of hypoxia and several indirect effects of pro-angiogenic factors, which could further increase the synthesis of VEGF. IL-1α has been shown to regulate VEGF expression in gastric cancer [30].

In an earlier study we demonstrated that enhancement of VEGF expression by IL-1α mediated through IL-1RI plays an important role in the metastatic and invasive behaviors of gastric cancer cells [15]. The results also suggested that I IL-1α has a significant function in tumor metastasis. In the present study, we evaluated tumor–stromal cell interactions mediated by IL-1RA. We first detected the expression of IL-1α, VEGF, and IL-1RI in gastric cancer cell lines. We then investigated whether IL-1α promotes VEGF secretion by human gastric cancer cells and human umbilical vein endothelial cells (HUVECs) and, if so, whether and how IL-1RA affects the proliferation, invasion, and angiogenesis of HUVECs. Our results show that IL-RA inhibits angiogenesis through the IL-1α/VEGF pathway in gastric cancer cell lines.

## Materials and method

### Materials and reagents

Recombinant human IL-1RA (rIL-1RA) was provided by Pepro Tech EC Ltd. (London, UK, and recombinant human IL-1α (rIL-1α) was purchased from Diaclone (Besancon, France).

### Cell lines and culture condition

Three kinds of gastric cancer cell lines derived from human gastric cancer were examined: MKN45, NUGC-4, and AGS. All cell lines were purchased from the American Type Culture Collection (Rockville, MD) and maintained in RPMI 1640 (Sigma Chemical Co., St. Louis, MO) supplemented with 10% heat-inactivated fetal bovine serum (FBS). HUVECs were obtained from Kurubo Co. (Osaka, Japan) and maintained in HuMedia-EG2 medium supplemented with 2% FBS, 5 ng/mL basic fibroblast growth factor, 10 ug/mL heparin, 10 ng/mL epidermal growth factor, and 1ug/mL hydrocortisone according to the supplier’s instructions (Kurubo Co.). All cells were incubated at 37 °C in a humidified atmosphere of 5% CO_2_ in air.

### Reverse transcription-PCR analysis of IL-1α, IL-1RI and VEGF mRNA expression

Total RNA was extracted from gastric cancer cells with an Isogen kit (Nippon Gene, Tokyo, Japan) and quantities were determined spectrophotometrically. Aliquots (5 μg) of total RNA pretreated with random hexamers and dNTP mix were incubated at 65 °C for 5 min, chilled on ice, then reverse-transcribed into cDNA in a cDNA Synthesis Mix containing 10 × RT buffer, 25 mM MgCl_2_, 0.1MDTT, RNaseOUT, and 200U SuperScript III RT (Invitrogen, San Diego, CA) at 50 °C for 50 min, followed by incubation at 85 °C for 5 min to terminate the reaction. Aliquots (1 μL) of the reaction mixture were used as templates for PCR analysis. Amplification reactions were performed in a DNA Thermal Cycler (Takara PCR Thermal Cycler MP Model TP3000; Takara Bio Inc., Otsu, Japan) using the primer sequences and PCR conditions shown in Table 1. Amplified DNA fragments were resolved by electrophoresis in 1.5% agarose gels containing ethidium bromide.

**Table 1:** Primer sequence and PCR cycling conditions

Gene name	Primer sequences	T_m_ (ºC)	Cycles (*n*)	Length (bp)	Accession number
Interleukin 1 alpha (IL-1α)	F: 5′-AATGACGCCCTCAATCAAAG-3′	54	35	206	NM-000575
	R: 5′-TGGGTATCTCAGGCATCTCC-3′				
Vascular endothelial growth factor (VEGF)	F: 5′-AAGGAGGAGGGCAGAATCAT-3′	54	35	226	NC-000006
	R: 5′-ATCTGCATGGTGATGTTGGA-3′				
Interleukin 1 receptor type I (IL-1RI)	F: 5′-GAAGACCCTCACCCTTACCC-3′	56	35	205	NM_001177704
	R: 5′-AAGGGACAACTTTGCGGTTC-3′				

### Real-time quantitative PCR

The PCR analysis was performed using a LightCycler apparatus (Roche Applied Science; Penzberg, Germany). Freshly isolated RNA was converted to cDNA using the PrimeScrip™ TR Regent kit (Takara Bio Inc.), and the PCR reaction was performed using the TaqMan^®^ Gene Expression Assay kit (Applied Biosystems, Foster City, CA). The IL-1α mRNA expression level is given as relative copy numbers normalized against glyceraldehyde 3-phosphate dehydrogenase (GAPDH) mRNA and shown as mean ± standard deviation. Relative IL-1α mRNA expression was calculated using the formula $\left( {A/G \div A_{0} /G_{0} } \right)$, where *A* is the relative copy numbers of IL-1α mRNA; *G* is the relative copy number of GAPDH mRNA; *A*_0_ and *G*_0_ are relative IL-1α and GAPDH mRNA expression levels from the standard cDNA dilutions as a nontemplate control.

### Enzyme-linked immunosorbent assay for IL-1α protein measurement

Both cells from the gastric cancer cell lines (MKN45, NUGC-4, AGS) and HUVECs were seeded at a density of 2 × 10^5^ cells/mL into 12-well plates containing medium supplemented with 10% FBS and cultured overnight, following which the medium in each well was replaced with the same medium supplemented with 1% FBS and the cells cultured for a further 48 h. Cell numbers were then determined, and the culture media were collected and centrifuged at 1500 rpm for 15 min to remove the pellets. The supernatants were stored at − 80 °C until used in the enzyme-linked immunosorbent assay (ELISA). The concentration of IL-1α in the supernatants (2 × 10^5^/mL cells) was measured using an ELISA kit (R&D Systems, Abingdon, UK) according to the manufacturer’s instructions.

### ELISA for VEGF protein

Similar to the determination of IL-1α protein, for VEGF protein measurement HUVECs and cells of the three gastric cancer cell lines (MKN45, NUGC-4, AGS) were seeded at a density of 2 × 10^5^/mL cells into 12-well plates and cultured overnight, following which the medium in each well was exchanged and the cells cultured for a further 48 h with or without the addition of rIL-1α (1, 10, 100 ng/mL) and IL-1RA (100 ng/mL) in the exchanged culture medium for HUVECs and with or without rIL-1RA (1, 10, 100 ng/mL) in the exchanged culture medium for cells of the gastric cancer lines. Cell numbers were the determined, and the culture media were harvested and microfuged at 1500 rpm for 15 min to remove the particles. The supernatants were frozen at − 80 °C until use in the ELISA. The concentration of VEGF in the supernatants per 2 × 10^5^/mL cells with or without stimulation from IL-1α and IL-1RA were measured using an ELISA kit (R&D Systems) according to the manufacturer’s instructions.

### HUVECs proliferation assay following rIL-1RA treatment

The HUVECs were seeded at a density of 2 × 10^4^ cells/100 uL into 96-well flat-bottomed plates and allowed to adhere overnight, following which the medium in each well was changed and the cells cultured further in unsupplemented medium (control) or in medium supplemented by different concentrations of rIL-1RA (1, 10, 100 ng/mL). The medium in the wells was exchanged every 24 h, and after 72 h of incubation, 10 uL WST-1 reagent was added to each well; the HUVECs were then incubated for 2 h at 37 °C. HUVEC proliferation was measured using the Premix WST-1 Cell Proliferation Assay System (Takara Bio Inc.) according to the manufacturer’s instructions. The absorbance was measured by microplate reader (Molecular Devices, Sunnyvale, CA) at a test wavelength of 450 nm and reference wavelength of 690 nm.

### Proliferation assay of gastric cancer cells treated with rIL-1RA

Cells of the gastric cancer cell lines MKN45, NUGC-4, and AGS were seeded at a density of 2 × 10^4^/100 uL cells into 96-well flat-bottomed plates and allowed to adhere overnight, following which the medium in each well was changed and the cells cultured further in unsupplemented medium (control) or in the medium supplemented by different concentrations of rIL-1RA (1, 10, 100 ng/mL). The medium in the wells was changed every 24 h, and after 72 h of incubation, 10 μL WST-1 reagent was added to each well; the gastric cancer cells were then incubated for 4 h at 37 °C. Cell proliferation was measured using the Premix WST-1 Cell Proliferation Assay system (Takara Bio Inc.) according to the manufacturer’s instructions.

### In vitro migration of HUVECs following pretreatment with rIL-1RA

The migration capability of HUVECs was determined using BioCoat Matrigel Invasion Chambers (Becton–Dickinson, Bedford, MA). This system consists of cell culture inserts containing a PET membrane coated with Matrigel Matrix that allows only invasive cells to migrate through the membrane to the other side. After rehydration for 2 h in a humidified incubator at 37 °C with 5% CO_2_, the HUVECs were seeded at a density of 1.0 × 10^5^ cells/mL into Matrigel pre-coated trans-wells containing 8-μm pore-size polycarbonate membranes, following which trans-wells chambers containing only basic medium (control) or medium pretreated with 1, 10, or 100 ng/mL IL-1RA were placed in 24-well plates. After a 24-h incubation, non-filtered cells were removed from the upper surface of membrane by gentle scrubbing with cotton-tipped applicators; the cells that passed through the membrane and invaded the opposite side of the wells were fixed with 70% ethanol, stained with Giemsa solution, and then counted in five random fields of the low filter surface under a microscope at ×200 magnification.

### Migration of HUVECs pretreated with co-cultured gastric cancer cells and rIL-1RA

To further investigate whether gastric cancer cell-derived IL-1α and rIL-1RA influence HUVEC migration capability, we performed the HUVEC migration assay using a double-chambers method. Gastric cancer cells (MKN45 and NUGC-4) were seeded at a density of 1 × 10^5^/mL cells into 24-well plates with or without 1, 10, or 100 ng/mL rIL-1RA; at the same time, trans-well chambers (containing 5 × 10^4^ HUVECs/mL per chamber) were plated into 24-well plates and allowed to incubate for 24 h. The numbers of invasive HUVECs were determined as described above.

### In vitro angiogenesis assay by treatment with rIL-1RA

To investigate the influence of rIL-1RA on tube formation by HUVECs, fibroblasts and HUVECs were co-cultured in the basal culture medium only or in basal culture medium containing rIL-1RA (1, 10 or 100 ng/mL) and assayed using an Angiogenesis Assay kit (Kurabo Co., Osaka, Japan) according to the manufacturer’s instructions. The media were exchanged every 3 days, and HUVECs and fibroblasts were co-cultured for a total of 11 days. Angiogenesis was detected by staining with anti-CD31 antibody according to the manufacturer’s protocols. The tube formation area was measured quantitatively over ten different fields for each condition using an image analyzer (Kurabo Co.).

### In vitro angiogenesis assay by treatment with gastric cancer cell co-culture

To investigate the effect of gastric cancer cells on angiogenesis by HUVECs, gastric cancer cell lines (MKN-45 and NUGC-4), HUVECs, and fibroblasts were co-cultured using a double-chamber method in 24-well plates. MKN-45 and NUGC-4 cells (1 × 10^5^ cells/mL) were seeded into trans-well chamber consisting of polycarbonate membranes with 0.45-µm pores and allowed to adhere overnight. The trans-well chambers were then placed into the HUVEC/fibroblast co-culture system and exchanged on day 1. The co-culture system was incubated for 12 days, and angiogenesis evaluated as described in the previous section. The assay allowed us to evaluate angiogenesis quantitatively and examine tumor–stromal cell interactions. In the same way, the effects of rIL-1RA on HUVEC tube formation in the presence of gastric cancer cells were also assessed.

### Statistical analysis

Statistical comparisons were performed using Student’s *t* test for paired observations and one-way analysis of variance with a post hoc test for multiple comparisons. Data are presented as the mean ± standard deviation. *P* < 0.05 was considered to be statistically significant. Each experiment was repeated three times and was carried out in triplicate.

## Results

### Expression of IL-1α, IL-1RI, and VEGF mRNA in gastric cancer cell lines

**Fig. 1:**
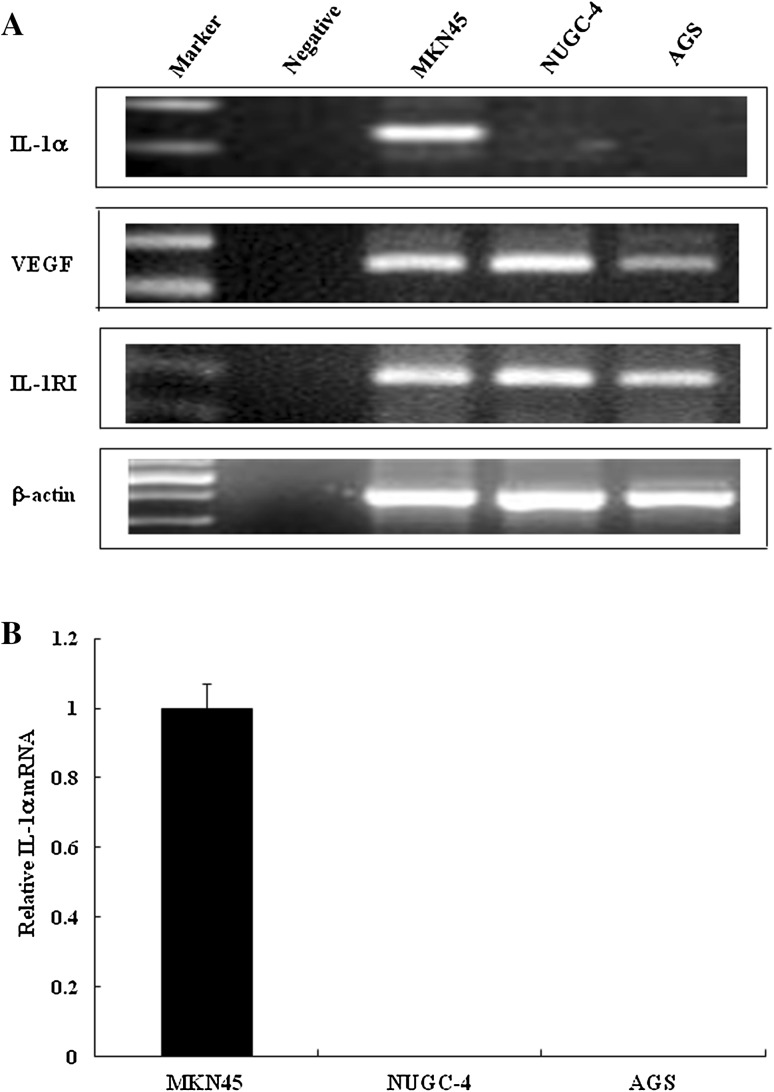
Expression levels of interleukin-1 alpha (*IL-1α*), interleukin 1 receptor type I (*IL-1RI*), and vascular endothelial growth factor (*VEGF*) mRNA in gastric cancer cell lines MKN45, NUGC-4, and AGS. **a** PCR products stained with ethidium bromide were subjected to 1.5% agarose gel electrophoresis. β-actin served as a loading control. **b** Relative expression of IL-1α mRNA in gastric cancer cell lines compared to glyceraldehyde 3-phosphate dehydrogenase (GAPDH) was assessed using semi-quantitative reverse transcription (RT)-PCR

The results of the reverse transcription (RT)-PCR analysis revealed that IL-1α mRNA was expressed only in the MKN45 cell line; no expression of IL-1α mRNA was detected in the NUGC-4 and AGS cell lines (Fig. 1a). Relative expression of IL-1α mRNA was determined by semi-quantitative RT-PCR, with the results agreeing with those of the RT-PCR experiment (Fig. 1b).

### Secretion of IL-1α and VEGF protein by gastric cancer cell lines

**Fig. 2:**
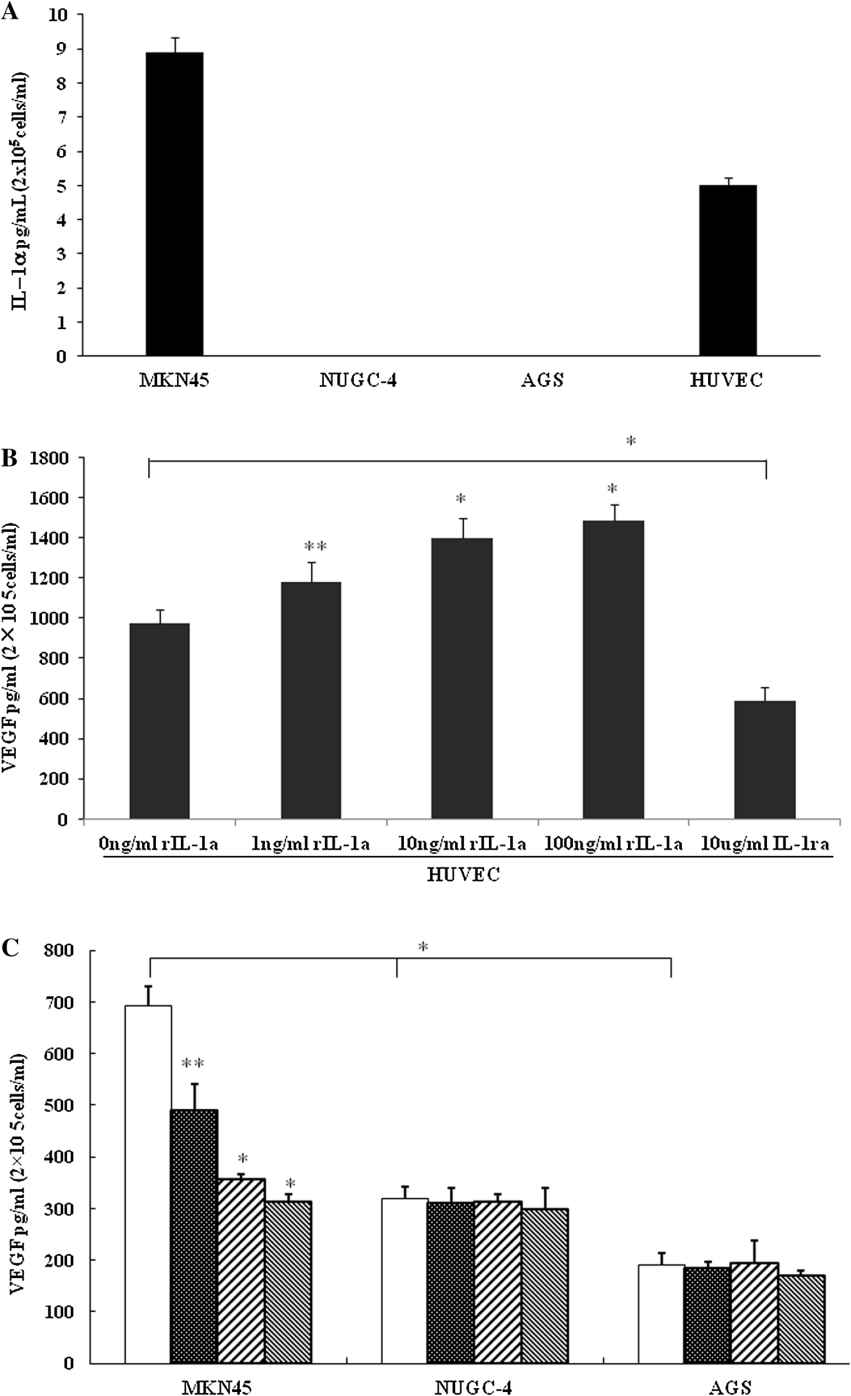
**a** Secreted IL-1α levels in human umbilical vein endothelial cells (*HUVECs*) and gastric cancer cell lines MKN45, NUGC-4, and AGS. **b** Effect of IL-1α and IL-1RA on the level of VEGF secreted by HUVECs. Secreted VEGF levels were determined in culture medium of HUVECS by enzyme-linked immunosorbent assay (ELISA). **c** Interleukin-1 receptor antagonist (*IL-1RA*) influences the secretion of VEGF in gastric cancer cell lines. White columns Cultured cells without rIL-1RA (control), black grid columns 1 ng/mL rIL-1RA, left diagonal striped columns 10 ng/mL rIL-1RA, right diagonal striped columns 100 ng/mL rIL-1RA. ** b**,** c** Asterisks indicate significant difference from control at **P < 0.05, *P < 0.01. Columns and whiskers Mean and standard deviation (SD), respectively

We detected IL-1α protein in the supernatants of cultured MKN45 and HUVEC cells (7.922 ± 0.525 and 5.231 ± 0.367 pg/mL/2 × 10^5^cells, respectively), but not in those of cultured NUGC-4 and AGS cells (Fig. 2a). IL-1α significantly enhanced VEGF secretion by HUVECs in a dose-dependent manner (*P < 0.01, **P < 0.05), while VEGF secretion by HUVECs was blocked by rIL-1RA (*P < 0.01; Fig. 2c). The secretion of VEGF protein by MKN45 cells was higher than that by NUGC-4 and AGS cells (*P < 0.01). VEGF secretion by MKN45 cells was blocked by rIL-1RA in a dose-dependent manner (compared with control, *P < 0.01, **P < 0.05), but that by NUGC-4 and AGS was not affected (Fig. 2b).

### Effect of IL-1RA on proliferation of HUVEC

**Fig. 3:**
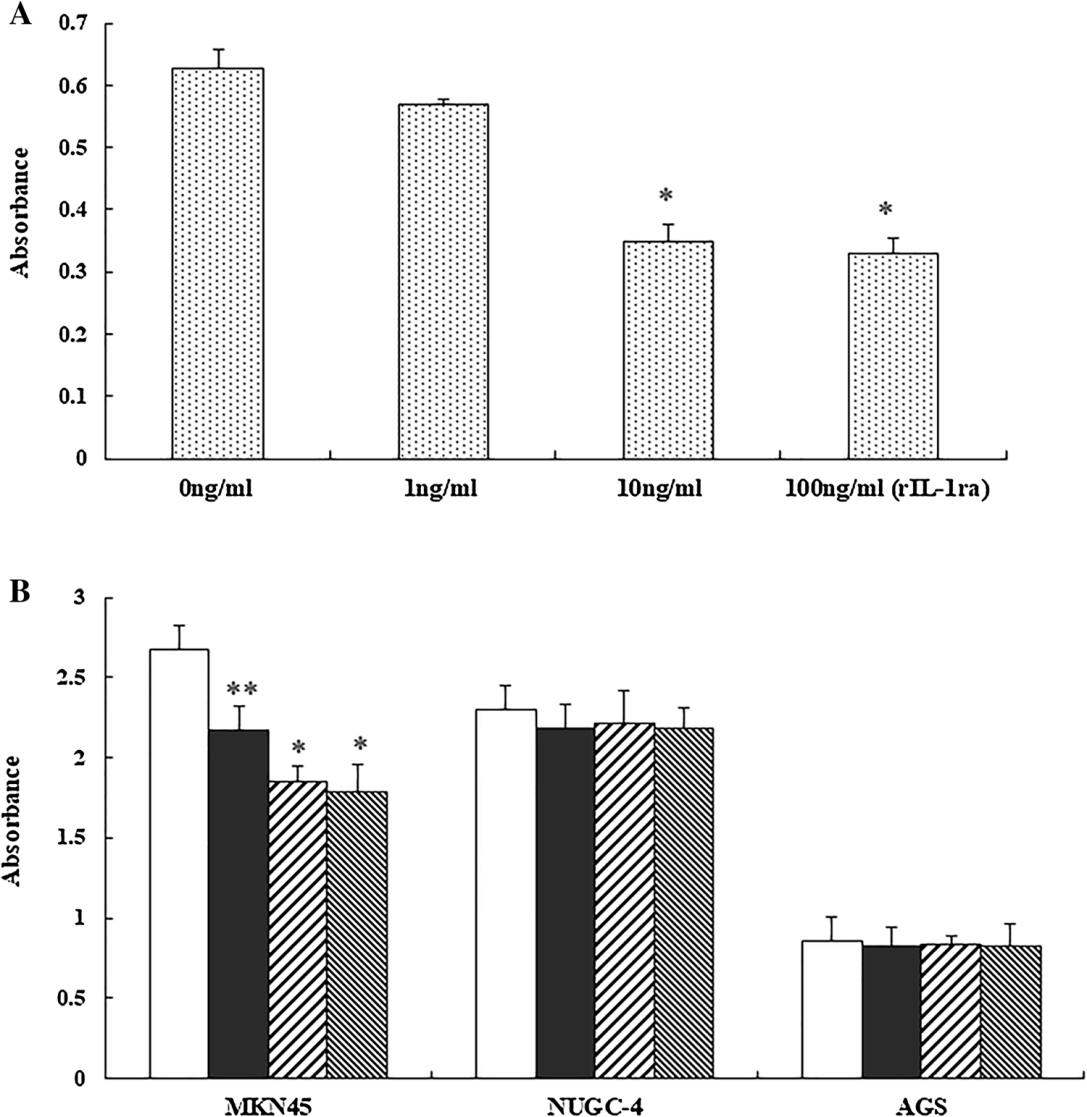
**a** Effect of IL-1RA on HUVEC proliferation. The premixed WST-1 Cell Proliferation Assay was used to measure the effect of recombinant human IL-1RA (rIL-1RA) on HUVEC proliferation. Absorbance was assessed at 450 and 690 nm and is presented as the mean (column) and SD (whiskers). One-way analysis of variance was used for multiple comparisons, followed by the Student–Newman–Keuls test. **b** Effect of rIL-1RA on gastric cancer cells proliferation compared with control (0 ng/mL rIL-1RA, white columns; 1 ng/mL rIL-1RA, black columns; 10 ng/mL rIL-1RA, left diagonal striped columns 10 ng/mL; 100 ng/mL rIL-1RA, right diagonal striped columns. Asterisks indicate significant difference from the control at *P < 0.01 or **P < 0.05. Columns and whiskers Mean and SD, respectively

The proliferation of HUVECs was inhibited by IL-1RA in a dose-dependent manner, with IL-1RA significantly decreasing the proliferation of HUVECs at a concentration of 10 and 100 ng/mL (compared with 0 and 1 ng/mL; *P < 0.01; Fig. 3a). IL-1RA not only inhibited HUVEC proliferation but also inhibited the proliferation of MKN45 gastric cancer cells in a dose-dependent manner (*P < 0.01, **P < 0.05 compared with 0 ng/mL); however, it did not affect the proliferation of NUGC-4 and AGS cells (Fig. 3b).

### Effect of IL-1RA or gastric cancer cell co-culture on HUVEC migration

**Fig. 4:**
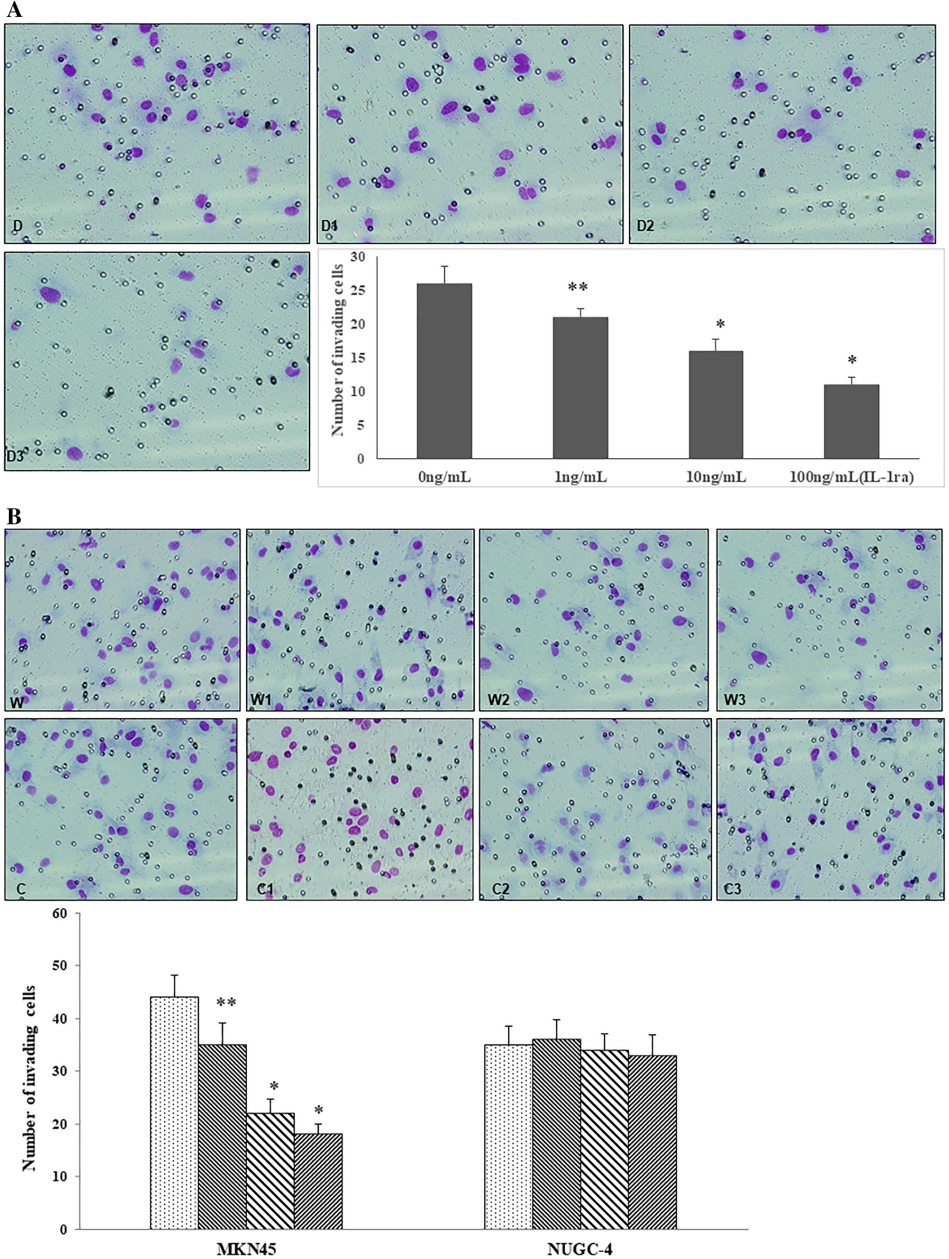
Effect of IL-1RA on HUVEC migration. **a** The influence of different concentrations of IL-1RA on HUVEC migration was assessed using the BD Bio-Coat Matrigel invasion assay system. D, 0 ng/mL IL-1RA; D1, 1 ng/mL IL-1RA, D2 10 ng/mL IL-1RA; D3, 100 ng/mL IL-1RA. **b** Effect of co-culture with gastric cancer cells (gastric cell lines MKN45 and NUGC-4) treated with no rIL-1RA (leftmost column) or different concentrations of rIL-1RA on HUVAC migration: columns with fine diagonal lines to the left 1 ng/mL rIL-1RA, columns with large diagonal lines to the left 10 ng/mL rIL-1RA, columns with fine diagonal lines to the right (rightmost column) 100 ng/mL rIL-1RA. W, Co-culture with MKN45; W1, with MKN45 + 1 ng/mL IL-1RA; W2, with MKN45 + 10 ng/mL IL-1RA; W3, with MKN45 + 100 ng/mL IL-1RA. C, Co-culture with NUGC-4; C1, with NUGC-4 + 1 ng/mL IL-1RA; C2, with NUGC-4 + 10 ng/mL IL-1RA; C3, with NUGC-4 + 100 ng/mL IL-1RA. Asterisks indicate significant difference from the control (0 ng/mL rIL-1RA) at *P < 0.01, **P < 0.05. Columns and whiskers Mean and SD

The addition of IL-1RA at 10 or 100 ng/mL to the culture media significantly inhibited HUVEC migration (*P < 0.01 compared to 0 ng/mL; Fig. 4a). In order to verify the interaction between gastric cancer cell-derived IL-lα and stromal cells in the tumor microenvironment, we determined the effect of IL-1RA by assessing the inhibition of gastric cancer cell-derived IL-1α and downregulation of HUVEC migration in a co-cultivation system consisting of gastric cancer cells and HUVECs. The migration capability of HUVECs was downregulated by IL-1RA in the MKN45 cell co-cultivation system (*P < 0.01, **P < 0.05), but no effect of IL-1RA was detected in the NUGC-4 co-cultivation system (Fig. 4b).

### Effect of IL-1RA on HUVEC tube formation

**Fig. 5:**
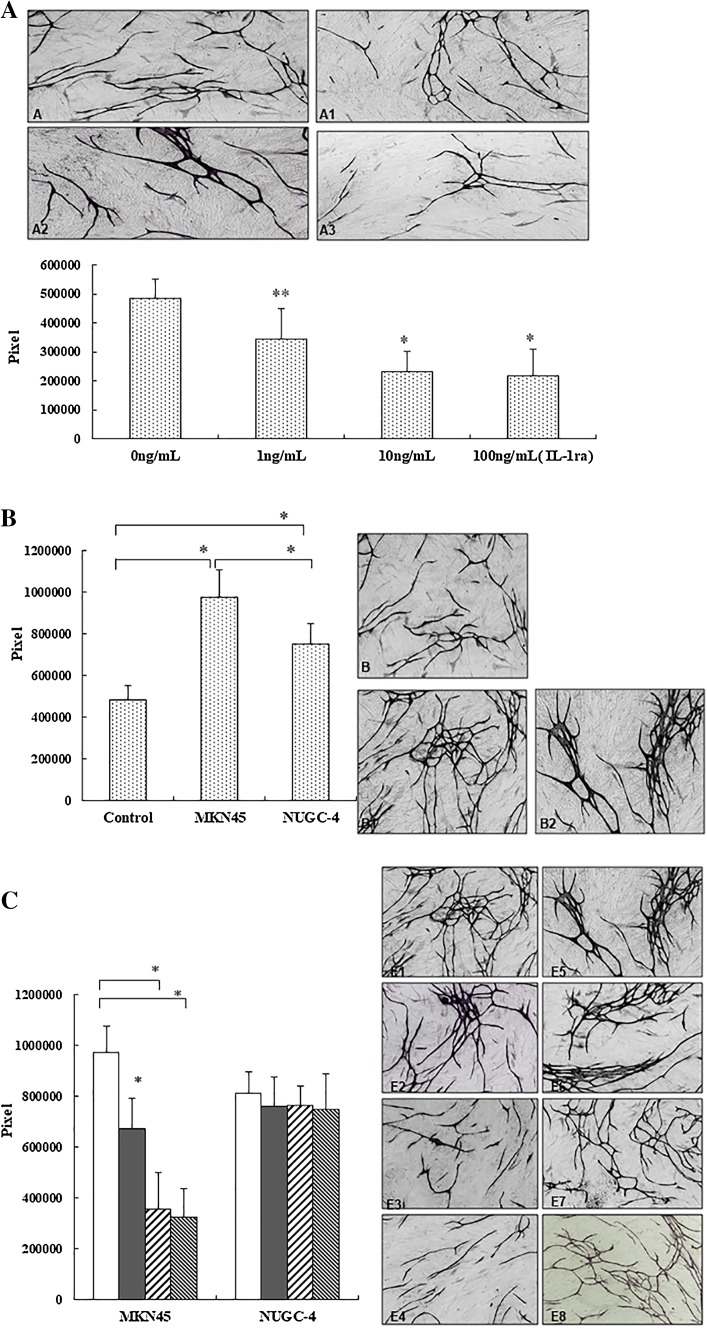
**a** Effect of IL-1RA on HUVEC tube formation. A, 0 ng/mL IL-1RA; A1, 1 ng/mL IL-1RA; A2, 10 ng/mL IL-1RA; A3, 100 ng/mL IL-1RA (×200). Columns Mean pixels of HUVEC tube formation area. Asterisks indicate significance at *P < 0.01 and **P < 0.05 vs. the control (0 ng/mL IL-1RA). **b** Effect of MKN45 and NUGC-4 on angiogenesis using the double chamber approach. Tube formation by HUVECs was significantly enhanced by co-culture with MKN45 cells compared with control or co-culture with NUGC-4. Asterisks indicate significant difference at *P < 0.01. B, Control; B1, co-culture with MKN45; B2, co-culture with NUGC-4. **c** Effect of IL-1RA on HUVEC tube formation in co-culture system. ×200 (0 ng/mL, white columns; 1 ng/mL, black columns; 10 ng/mL, left diagonal striped columns 10 ng/mL; 100 ng/mL, right diagonal striped columns). E1, Co-culture with MKN45; E2, with MKN45 + IL-1RA (1 ng/mL); E3, with MKN45 + rIL-1RA (10 ng/mL); E4, with MKN45 + rIL-1RA (100 ng/mL). E5, Co-culture with NUGC-4; E6, with NUGC-4 + IL-1RA (10 ng/mL); E7, with NUGC-4 + rIL-1RA (10 ng/mL); E8, with NUGC-4 + rIL-1RA (100 ng/mL). Asterisks indicate significant difference at *P < 0.01. Columns and whiskers Mean and SD

Tube formation by HUVECs was significantly inhibited by the treatment with IL-1RA in a dose-dependent manner (**P < 0.05, *P < 0.01 compared with 0 ng/mL IL-1RA) (Fig. 5a). Tube formation by HUVECs was significantly enhanced by co-culture with MKN45 cells compared with HUVECs grown without cancer cells (control) or with NUGC-4 cells (*P < 0.01, **P < 0.05; Fig. 5c). The enhanced angiogenesis in co-culture with MKN45 cells was inhibited by the addition of IL-1RA in a dose-dependent manner (compared with control, *P < 0.01) (Fig. 5D).

## Discussion

The results of this study provide the novel insight that there is a synergistic interaction between gastric cancer cells and endothelial cells in terms of the biological effects of cytokines in the tumor microenvironment. The tumor microenvironment consists of tumor, immune, stromal, and inflammatory cells, all of which produce cytokines, growth factors, and adhesion molecules that may promote tumor progression and metastasis [30, 31]. All cells and cytokines that interact closely with each other play an important role in inflammatory and angiogenic processes and tumor cell proliferation. Interestingly, an association between chronic inflammation and tumor development or progression has been reported, with approximately 15% of all cancers attributed to inflammatory etiologies [32]. IL-1α, one of the most potent inflammatory cytokines, is reported to increase gastric cancer metastasis [33].

Gastric cancer is a very common neoplasm that has a high propensity for liver metastases, which in turn leads to a poor prognosis for patients. There is currently no highly effective approaches for the treatment of advanced or metastatic gastric cancer; consequently, there is an urgent need for new non-surgical treatment strategies [34, 35]. We have explored the mechanisms of liver metastasis in gastric cancer and how to inhibit or retard its progression with reasonable methods. Our study shows that IL-1α stimulates endothelial cell proliferation and migration and enhances tube formation in a concentration-dependent way through the IL-1α VEGF signaling pathways [31]. In the present study, we detected the expression of IL-1α, both the mRNA and secreted protein, only in gastric cancer cell line MKN45, but not in gastric cancer cell lines NUGC-4 and AGS. Secreted IL-1α protein was also found in the supernatant of cultured HUVECs. Similarly, previous studies have shown that liver metastasis of gastric cancer is regulated by IL-1α via the enhanced proliferation of cancer cells and the accelerated process of angiogenesis via enhanced tube formation by HUVECs [31]. These results indicate that the increased expression of IL-1α is closely related to high liver metastasis, but not to low liver metastasis, in gastric cancer cell lines. Furthermore, increased IL-1α expression may enhance the metastatic potential of gastric cancer cells. Therefore, we hypothesize that IL-1α from highly metastasized gastric cancer cells enhance liver metastasis by regulating tumor progression and angiogenesis.

Angiogenesis, the process by which new blood vessels are formed from preexisting vessels, is essential for the growth and progression of the solid tumor. A large number of growth factors regulate angiogenesis, among which VEGF is the most important. Through a range of signaling processes that stimulate the growth, migration, survival, and permeability of vascular endothelial cells, the VEGF pathway is able to trigger various functions, thereby activating the process of neovascularization by which endothelial cells arise from progenitor cells and new blood vessels sprout from old ones. The expression of VEGF mRNA has been reported to correlate with poor prognosis and liver metastasis in primary gastric cancer [10, 26, 36]. The results of ELISA in our experiment demonstrated that the level of secreted VEGF correlated with the liver metastasis potential of gastric cancer cell lines, with relatively higher levels of VEGF secreted by the highly metastatic liver cell line MKN45 compared with the low metastatic gastric cancer cell lines NUGC-4 and AGS. Interestingly, VEGF secretion by HUVEC and MKN45 cells was blocked by IL-1RA in a dose-dependent manner.

In our study, we explored the interactions between HUVECs, IL-1RA, and gastric cancer cell lines with different liver metastasis potential. The proliferation assay indicated that IL-1RA not only inhibited HUVEC proliferation but also inhibited the proliferation of gastric cancer cells, both in a dose-dependent manner (*P* < 0.01). Supernatants from cultured MKN45 cells significantly enhanced HUVEC proliferation. This enhanced proliferation of HUVECs was inhibited by IL-1RA (*P* < 0.01) but failed to affect the response of HUVECs to the supernatants of cultured NUGC-4 and AGS cells. These data show that the IL-1α produced by gastric cancer cells could promote the proliferation of HUVEC and that IL-1RA inhibited the proliferation of HUVECs. The presence of IL-1RA significantly inhibited the migration of HUVECs in a dose-dependent manner (*P* < 0.01). We next examined the effect of IL-1RA by assessing the inhibition of gastric cancer cell-derived IL-1α and downregulation of HUVEC migration in a co-cultivation system consisting of gastric cancer cells and HUVECs. The migration capability of HUVECs was downregulated by IL-1RA in the MKN45 cell co-cultivation system. but there was no effect of IL-1RA in the NUGC-4 co-cultivation system. In the angiogenesis assay, we found that tube formation by HUVECs was significantly inhibited by treatment with IL-1RA in a dose-dependent manner. Tube formation by HUVECs was significantly enhanced by co-culture with MKN45 cells. The addition of IL-1RA was able to inhibit the enhanced angiogenesis in the presence of MKN45 cells. These results indicate that those gastric cancer cells with high liver metastatic potential also have a high angiogenic potential and that tumor cell-derived cytokines play an important role in angiogenesis and metastasis. HUVECs secreted IL-1α, and IL-1RA could down regulate the proliferation, migration, and angiogenesis of HUVECs by inhibiting the IL-1α and VEGF signaling pathways.

In conclusion, the results of our experiments indicate that IL-1α and VEGF are important molecules in the interaction between gastric cancer cells and the tumor microenvironment and that IL-1α expressed by high liver metastatic gastric cancer cells enhances the secretion of VEGF in an autocrine manner. In turn, these factors enhance the metastatic potential of gastric cancer. Our findings also show that IL-1RA can inhibit tumor cell-dependent angiogenesis. These data suggest that IL-1RA may be a potential target in the clinical treatment of gastric cancer patients, possibly alone or in combination with an anti-VEGF antibody or with other chemotherapy agents. In addition, our work should encourage further study into more potential angiogenic regulators in oncology.
